# *In Situ* Expression of Regulatory Cytokines by Heart Inflammatory Cells in Chagas' Disease Patients with Heart Failure

**DOI:** 10.1155/2012/361730

**Published:** 2012-07-03

**Authors:** Denise Bertulucci Rocha Rodrigues, Marlene Antonia dos Reis, Audrey Romano, Sanívia Aparecida de Lima Pereira, Vicente de Paula Antunes Teixeira, Sebastião Tostes Junior, Virmondes Rodrigues

**Affiliations:** ^1^Laboratory of Immunology, Institute of Biological Sciences, Federal University of Triângulo Mineiro, 38125-180 Uberaba, MG, Brazil; ^2^Departamento de Imunologia e Patologia, University of Uberaba, 38055-500 Uberaba, MG, Brazil; ^3^INSERM-U906, 13385 Marseille, France; ^4^Laboratory of Immunology, Federal University of Triângulo Mineiro, 38125-180 Uberaba, MG, Brazil

## Abstract

Chagas' disease is caused by the protozoan parasite *Trypanosoma cruzi*. The immune system plays an important role in the reduction of parasite load, but may also contribute to the development of lesions observed during the chronic phase of the disease. We analyzed cytokines produced by inflammatory heart cells in 21 autopsy samples obtained from patients with Chagas' disease divided according to the presence or absence of heart failure (HF). Left ventricular sections were analyzed by immunohistochemistry using antibodies against human IL-4, IFN-**γ**, TGF-**β**, TNF-**α**, and NOS2. *In situ* mRNA expression was quantified by a Low Density Array. The number of IFN-**γ**-positive cells was significantly higher than IL-4 positive cells. TNF-**α**, TGF-**β** and NOS2 were detected in 65%, 62% and 94% of samples respectively. There was an association between TNF-**α**-producing cells and the presence of HF. Subjects with HF presented higher levels of STAT4 mRNA, whereas FoxP3 and STAT6 levels were similar in the two groups. A Th1 cytokine pattern predominated in the cardiac inflammatory cell infiltrate of Chagas' disease patients associated with HF. High degree of fibrosis was associated with low NOS2 expression. These results support the idea that Th1 immune responses are involved in heart lesions of Chagas' disease patients.

## 1. Introduction

Chagas' disease is caused by the protozoan parasite *Trypanosoma cruzi*, which is transmitted to humans by contact with the feces of blood-sucking triatomine insects, transfusion of blood or blood derivatives, and organ transplantation. According to the WHO estimates, 16–18 million people were infected in the Americas in the 1980s [1]. Although transmission is controlled in some countries, the infection persists and each year a large number of patients in the chronic phase develop symptoms [2].

During the chronic phase, a long period of latency, called the indeterminate phase which lasts several years or throughout life, is observed in approximately 60% of patients with Chagas' disease. Clinical manifestations that result in heart and/or digestive organ damage occur in the remaining 40% of patients at different intensities, alone or in association [2, 3]. During this phase, parasites are rarely detected in peripheral blood and the diagnosis is based on the presence of parasite-specific antibodies [4].

Heart complications are the major cause of morbidity and mortality in Chagas' disease patients. Differences in the degree of damage to the conduction system are observed, and heart failure occurs among more severe cases [2]. Myocarditis can affect patients during the chronic phase, irrespective of clinical manifestations, although the degree of myocarditis and fibrosis that follows the inflammatory process is more intense in patients with heart failure [5, 6]. The inflammatory process observed in Chagas' disease is mainly characterized by the presence of CD8 and CD4 T lymphocytes and macrophages [7]. The presence of activated CD8 T lymphocytes has been demonstrated [8]. The extent and nature of the inflammatory reaction contribute to tissue damage and reduce cardiac function. These events lead to heart failure which is observed in severe cases of chronic Chagas' disease.

The balance of the T helper cell subpopulation has been studied in experimental models of *T. cruzi* infection and the results show that the Th1 subpopulation plays a role in the mechanism of parasite control [9, 10]. In this respect, IFN-*γ* and TNF-*α* synergistically act on NOS2 transcription and on the production of high levels of nitric oxide, which exhibits strong antiparasitic effects [9, 11, 12]. These cytokines have been shown to play a role in parasite control and also contribute to tissue damage [13, 14]. On the other hand, IL-4, a prototype Th2 cytokine, is associated with an increase of parasitemia. In humans, most data regarding the T helper cell balance is limited to the analysis of PMBC immune responses [15] and CD4 and CD8 cell infiltration [16, 17].

To address the nature of inflammatory cells in heart tissue and possible implications in the pathogenesis of heart failure in Chagas' disease, we investigated the number of cells expressing IFN-*γ*, TNF-*α*, IL-4, TGF-*β*, and NOS2 and the levels of FoxP3, STAT4, and STAT6 mRNA in heart tissues of subjects who had died during the chronic phase of the disease. To our knowledge, this is the first study investigating T helper cell subpopulation in heart tissue. The results demonstrated that the production of all mediators mentioned above and that the number of cells producing IFN-*γ* were higher than that producing IL-4. Moreover, large numbers of TNF-*α*-producing cells and high levels of STAT4 mRNA were associated with the occurrence of heart failure.

## 2. Materials and Methods

### 2.1. Patients

Twenty-one specimens of the left ventricular wall were obtained at autopsy of subjects with Chagas' disease who had died at the General Hospital of Triângulo Mineiro Federal University, Uberaba, MG, Brazil. The autopsies were performed within 2 to 6 hours after death, and two samples were collected from the midportion of the lateral wall of the left ventricle. One specimen was immediately fixed in buffered formalin, and the other was frozen in liquid nitrogen. After embedding in paraffin, serial 5 *μ*m sections were transferred to glass slides. Frozen samples were used in the low density array (LDA). All specimens were obtained from patients who had a positive reaction to anti-*T. cruzi* antibodies and who were considered to be in the chronic cardiac phase. Heart involvement affected only the electrical conduction system in 10 patients. In the remaining 11 subjects, the clinical records indicated heart failure, such as leg edema, and chest radiographs demonstrated global heart enlargement [2, 6]. Samples obtained from three healthy subjects who died in car accidents were used as controls in the LDA assays. The procedures were approved by the Ethics Committee of the Federal University of Triângulo Mineiro.

### 2.2. Histopathology and Quantification of Fibrosis

Fixed tissues were dehydrated and embedded in paraffin. Sections (5 *μ*m) were stained with hematoxylin-eosin (HE) and analyzed by light microscopy.

Morphometric analysis of fibrosis was performed using the KS300 automatic image-analyzing system (Kotron Electronic, Munich, Germany). Fibrous connective tissues were evaluated by picrosirius red staining, and the results are expressed as percentage of fibrosis area [18]. For further analysis, patients were empirically divided according to fibrosis intensity into low-grade fibrosis (up to 4%) and high-grade fibrosis (>4%).

### 2.3. Immunohistochemistry

For immunohistochemistry, deparaffinized sections were treated with 3% hydrogen peroxide in methanol for 10 min and incubated for 30 min at 90°C for antigen detection. The sections were incubated in 2% bovine serum albumin for 30 min at room temperature to reduce nonspecific binding. Next, the sections were individually incubated with anti-cytokine monoclonal antibodies specific for human IL-4 (1 : 100) (R&D, Minneapolis, MN, USA), TNF-*α* (1:200) (R&D); TGF-*β* (1 : 100) (R&D), NOS2 (1 : 100) (Santa Cruz Biotech, Santa Cruz, CA, USA), and IFN-*γ* (1 : 200) (Genzyme, Cambridge, MA, USA). All antibodies were diluted in 2% bovine serum albumin prior to use and incubated with the samples for 2 hours at 37°C. For the secondary antibody, the sections were incubated with biotinylated anti-mouse Ig, anti-rabbit Ig, and anti-goat Ig from Link System 002488 (Dako, Carpinteria, CA, USA) for 30 min at 37°C. After washing, the sections were incubated with streptavidin-peroxidase conjugate (Dako) for 30 min. The reaction was developed with diaminobenzidine (Sigma, St. Louis, MO, USA). The sections were counterstained with hematoxylin.

For histopathological analysis, the number of cells positive for each cytokine was counted in 20 fields at 400X magnification. The number of cells in each field and the area of each field (0.091575 mm^2^) were determined. The density of positive cells is expressed as the number of cells per mm^2^.

### 2.4. Low Density Array

After cell harvest, RNA was extracted with the TRIzol reagent (Invitrogen, Grand Island, NY, USA) according to manufacturer instructions. RNA purity and integrity were assessed with the Agilent 2100 bioanalyzer using the RNA 6000 Nano LabChip reagent set (Agilent Technologies, Santa Clara, CA, USA). RNA was quantified spectrophotometrically and then stored at −80°C. cDNA was synthesized using the High-Capacity cDNA Archive Kit (Applied Biosystem, Carlsbad, CA, USA). The master mixture contained 1X reverse transcription buffer, 1X deoxynucleotide triphosphate mixture, 1 unit/*μ*L RNase inhibitor, 1 unit/*μ*L MultiScribe Reverse Transcriptase, and 1X random primers. One *μ*g of total RNA was diluted in sterile water to a final volume of 100 *μ*L. The reaction mixture was incubated at 25°C for 10 min, followed by heat inactivation of the enzyme at 37°C for 120 min cDNA was stored at −20°C. Next, 2 *μ*L single-stranded cDNA (corresponding to 100 ng total RNA) was diluted in 98 *μ*L nuclease-free water and 100 *μ*L TaqMan Universal PCR Master Mix, and 100 *μ*L of the sample-specific PCR mixture was loaded into the sample port of Micro Fluidic Cards. The cards were then centrifuged twice for 1 min at 1200 g and sealed to prevent well-to-well contamination. The cards were placed in the Micro Fluidic Card Sample Block of an ABI Prism 7900 HT Sequence Detection System (SDS Software 2.1, Applied Biosystems). The thermal cycling conditions were 2 min at 50°C and 10 min at 94.5°C, followed by 40 cycles at 97°C for 30 s and at 59.7°C for 1 min. Each Micro Fluidic Card has a unique barcode, and Sequence Detection System plate documents store information about plate type, detector, sample/target gene configurations, thermal cycling conditions, data collection, and raw fluorescence data during each cycle. Micro Fluidic Cards were analyzed with RQ documents and the RQ Manager Software for automated data analysis. Experiments for three different donor cells and one healthy control, carried out in duplicate, were analyzed together as 1 relative quantity (RQ) study. Expression values of the target genes were normalized to the concentration of 18S rRNA. Gene expression values were calculated by the comparative threshold cycle (Ct) method, in which RNA samples from the control subject were designated as calibrators. In short, the Ct data for all human genes tested and 18S rRNA in each sample were used to create ΔCt values (Ct_experimental_ − Ct_18S rRNA_). Thereafter, ΔΔCt values were calculated by subtracting the ΔCt of the calibrator from the Ct value of each target. RQ was calculated using the equation: RQ = 2^−ΔΔCt^. The Micro Fluidic Cards detect a two-fold difference in gene expression at a confidence level of 99.7%. 

### 2.5. Statistical Analysis

Statistical analysis (unpaired *t*-test and Mann-Whitney test) was performed using the StatView software. Correlations between the numbers of cells positive for each cytokine were analyzed using the Spearman (*rS*) and the Pearson (*r*) correlation coefficients. Differences found were considered to be significant at *P* < 0.05.

## 3. Results 

### 3.1. Histopathology, Fibrosis Quantification, and Cytokine Expression

Myocardial samples from 21 chronic chagasic subjects were examined. Eleven patients had chronic heart failure, and 10 subjects did not present any previous clinical or anatomopathological signs of heart failure due to Chagas' disease. These samples were also classified according to fibrosis intensity.

**Figure 1:**
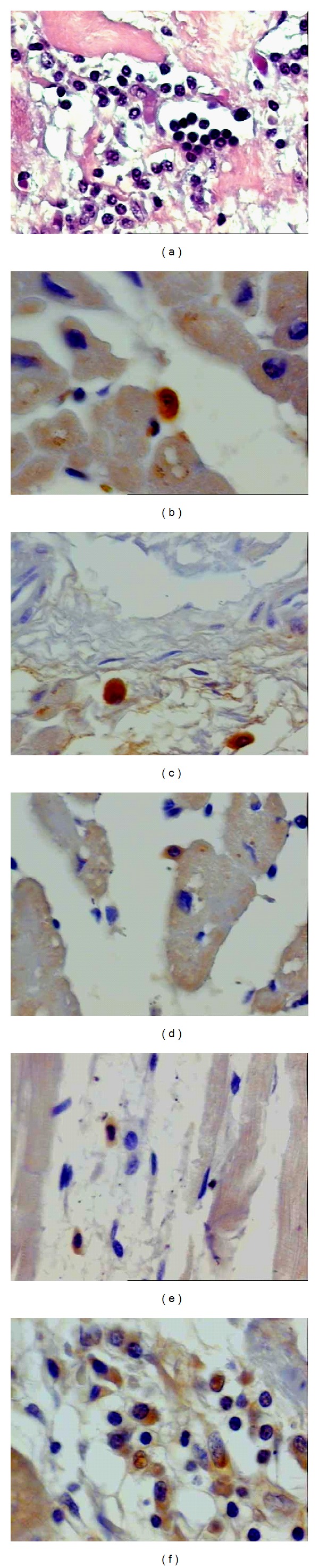
Histological sections of heart tissues obtained at autopsy from subjects with chronic chagasic cardiomyopathy. (a) Cellular exudation with a predominance of mononuclear cells around the fibrosis area (1,280X); (b) positive TNF-*α* staining and leukocytes in close contact with the myocardiocyte (1,280X); (c) positive IFN-*γ* staining in the inflammatory exudate (1,280X); (d) positive IL-4 staining in the inflammatory exudate and leukocytes in close contact with the myocardiocyte (600X); (e) discrete TGF-*β* immunostaining in the inflammatory exudate (1,280X); (f) positive NOS2 staining in the inflammatory exudate (1600X).

**Figure 2:**
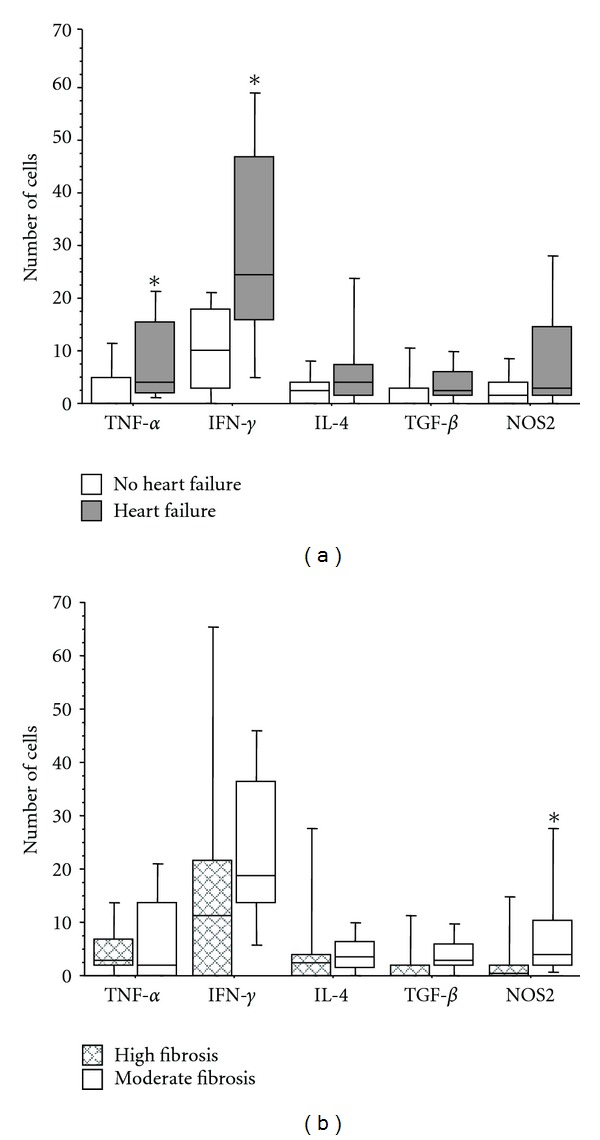
Number of inflammatory cells expressing cytokines and NOS2 in heart tissue from patients in the chronic phase of Chagas' disease. (a) Subjects were divided according to the presence (gray bar) or absence of heart failure (open bar). (b) Subjects were divided according to the presence of a high degree (hatched bar) or low degree of fibrosis (open bar). Horizontal lines represent the median, boxes represent the 25th to 75th percentiles, and vertical lines indicate the 10th to 90th percentiles. *P < 0.05 (Mann-Whitney test).

An inflammatory mononuclear cell infiltrate was observed in most cases inside and around the fibrosis area. The intensity of the inflammatory reactions was higher in subjects with heart failure. The panels in Figure 1 illustrate the inflammatory reaction and the immunohistochemical results. A significant association was observed between TNF-*α*-positive cells and the presence of heart failure (*P* = 0.040). TNF-*α*-positive cells were observed in 10 of the 11 cases with heart failure, with a median of 4 immunostained cells (range: 0 to 22 cells/mm^2^), and in 4 of the 10 cases without heart failure (Figure 2(a)).

**Figure 3:**
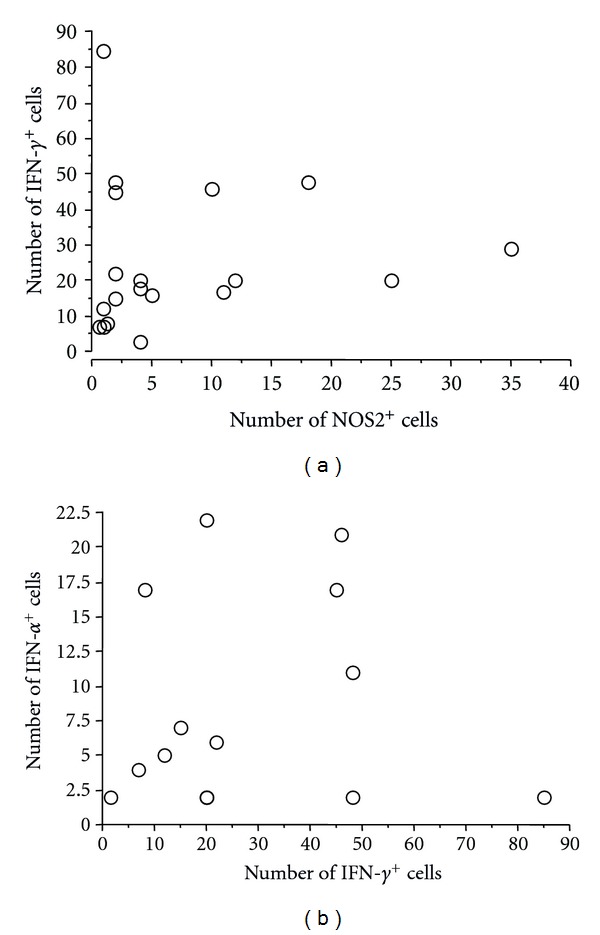
Correlation between the number of IFN-*γ*- and NOS2-immunostained inflammatory cells (a) and the number of TNF-*α*- and IFN-*γ*-immunostained inflammatory cells in 21 subjects with chronic chagasic cardiopathy (b). *P < 0.05 (Spearman's correlation).

There was a positive association between IFN-*γ*-producing cells and heart failure (*P* = 0.032). IFN-*γ*-positive cells were present in 8 of the 11 cases with heart failure, with a median of 24 immunostained cells (range: 0 to 85 cells), and in 7 of the 10 cases without heart failure (Figure 2(a)). TNF-*α* and IFN-*γ* are cytokines involved in the induction of NOS2 and have been implicated in the control of intracellular parasite growth and tissue damage. Although TNF-*α* and IFN-*γ* were associated with heart failure, no significant correlation was observed between NOS2-positive cells and heart failure. Cells positive for NOS2 were present in 10 of the 11 cases with heart failure and in 6 of the 10 cases without heart failure (Figure 2(a)). Moreover, there was no significant association between the presence of cells positive for the anti-inflammatory cytokines, IL-4 and TGF*β*, and the occurrence of heart failure (Figure 2(a)). On the other hand, a positive correlation was observed between the number of IFN-*γ*-positive and NOS2-positive cells, irrespective of the occurrence of heart failure (*P* = 0.02) (Figure 3(a)). There was also a positive correlation between the number of cells positive for IFN-*γ*and TNF-*α* (*P* = 0.03) (Figure 3(b)).

The intensity of fibrosis was also analyzed and was classified into low (up to 4% of total area) or high (>4%). Interestingly, the occurrence of intense fibrosis was associated with small numbers of NOS2-positive cells, suggesting that nitric oxide may protect against the development of fibrosis (Figure 2(b)). Fibrosis was not associated with any of the other cytokines tested (Figure 2(b)).

### 3.2. Th1/Th2 Cytokine Balance

**Figure 4:**
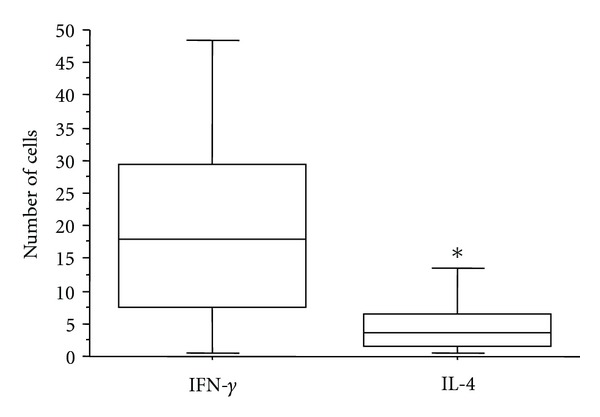
Number of IFN-*γ*- and IL-4-immunostained inflammatory cells in 21 subjects with chronic chagasic cardiopathy. Horizontal lines represent the median, boxes represent the 25th to 75th percentiles, and vertical lines indicate the 10th to 90th percentiles. *P < 0.05 (Mann-Whitney test).

**Figure 5:**
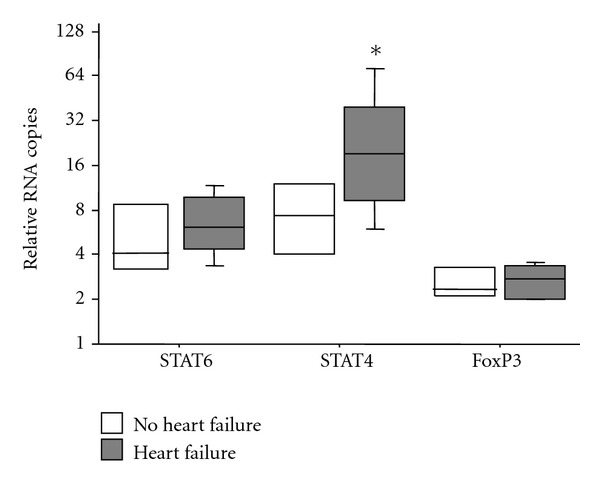
Relative number of mRNA copies in heart tissue of patients in the chronic phase of Chagas' disease according to the presence (gray bar) or absence of heart failure (open bar). Horizontal lines represent the median, boxes represent the 25th to 75th percentiles, and vertical lines indicate the 10th to 90th percentiles. *P < 0.05 (Mann-Whitney test).

The numbers of IFN-*γ*- and IL-4-positive cells were compared for the evaluation of the balance between Th1 and Th2 cytokine patterns. The number of IFN-*γ*-positive cells was significantly higher than that of IL-4-positive cells (*P* = 0.02), irrespective of the occurrence of heart failure, indicating that *T. cruzi* induced myocarditis by eliciting a Th1-like response (Figure 4). Furthermore, LDA analysis revealed significantly higher STAT4 levels in patients with heart failure compared to those without heart failure. Moreover, STAT4 levels were significantly higher than STAT6 levels in the heart failure group (Figure 5). These data suggest that heart failure in Chagas' disease is associated with a Th1 immune response. No significant differences between groups were observed for the other cytokine genes tested.

## 4. Discussion

In this study, we report the results of the expression of cytokines in heart tissue obtained from the left ventricular wall of subjects with Chagas' disease. The autopsies were performed within 2 to 6 hours after death, and the specimens were immediately frozen in liquid nitrogen or fixed in paraformaldehyde in phosphate buffer. Clinical involvement of the heart was analyzed considering previous clinical data and anatomopathological features observed during autopsy. 

TNF-*α* can induce collagen synthesis and fibrosis [19]. A previous study demonstrated that TNF-*α* gene polymorphisms and high expression of this cytokine were associated with human infection with *T. cruzi* [20]. In the present study, a positive association was observed between heart failure and fibrosis. In this respect, the loss of contractile cells and their replacement by fibrotic tissue contribute to heart failure. TNF-*α* may be part of this phenomenon, inducing tissue damage and fibrosis development. Other studies have shown TNF-producing cells in association with areas of tissue damage in acute models of *T. cruzi* infection [14]. In addition, the presence of this cytokine has been constantly observed in histopathological studies of hearts from subjects who had died of Chagas' disease [8]. TNF-*α* may also contribute to the development of heart failure through apoptosis and the induction of NOS2, producing nitric oxide which exerts strong negative inotropic effects [21–25]. TNF-*α* and IFN-*γ* act synergistically on NOS2 expression and the subsequent induction of death of the parasite [9]. In the present study, IFN-*γ* was positively associated with heart failure. Considering that IFN-*γ* has antifibrotic properties and TNF-*α* is involved in fibrosis, the synergistic effect of the two cytokines on NOS2 expression may have contributed to the development of heart failure. These results suggest that cytokines involved in parasite control, such as TNF-*α* and IFN-*γ* and the major mediator of parasite death, nitric oxide, may persist and induce mechanisms of tissue damage that contribute to the development of heart failure.

This study clearly demonstrated the predominance of a Th1 immune response in the inflammatory reaction seen in the heart of subjects with severe forms of Chagas' disease. The local production of IFN-*γ* is functional, since STAT4 mRNA was overexpressed in subjects with heart failure. Furthermore, IFN-*γ*-induced genes are upregulated in heart samples from patients with Chagas' disease [26]. Studies have shown a protective role of CD4+ T lymphocytes and IFN-*γ* responses in anti-*T. cruzi* immunity in murine models of Chagas' disease [27–30]. Conversely, studying chronic human infection with *T. cruzi*, other investigators demonstrated the production of higher levels of IFN-*γ* by PBMC from cardiac patients when compared to asymptomatic subjects and associated this production with pathogenesis [31, 32].

In conclusion, the present results show that the heart infiltrating T cells in Chagas' disease mainly have a Th1 phenotype. Severe heart involvement leading to heart failure seems to be multifactorial and associated with the presence of IFN-*γ*- and TNF-*α*-producing cells. Continuous antigen stimulation may help sustain the inflammatory response, with the intensity and pattern of this response promoting tissue damage and heart cell dysfunction that lead to heart failure.
